# Legends of p53: untold four-decade stories

**DOI:** 10.1093/jmcb/mjz079

**Published:** 2019-08-20

**Authors:** Hua Lu

**Affiliations:** Department of Biochemistry & Molecular Biology and Tulane Cancer Center, Tulane University School of Medicine, New Orleans, LA 70112, USA

Since its discovery in 1979, p53 has been the most intensively studied molecule in biomedical research and described as the most important tumor suppressor in human cancer. The more we study p53 the less we know about it as depicted by the cover of this issue. To celebrate the 40th anniversary of p53 discovery, to cheer researchers who have dedicated their entire careers to study p53, and to help readers better understand p53 and its history, this special issue of *Journal of Molecular Cell Biology* (JMCB) has fortunately collected remarkably interesting and historical papers from a number of hard core p53 researchers in the field of cancer, many of whom are pioneering and world-class scientists.

This issue is generally divided into three sections: Retrospect, Review, and Perspective. It also includes the opening remarks at the recent JMCB Symposium 2019, with the theme ‘The Legend p53 vs. Cancer’, as an Editorial and a meeting summary for this symposium.

For the Retrospect section, we have collected several essays about different stories at different stages of p53 discovery and research as well as scientific career development. These stories unveil the enigmatic history of p53, such as how p53 becomes a tumor suppressor from an oncogenic protein after a decade of its research and how its initially recognized oncogenic function is dramatically related to the later realized gain of function (GOF) of hotspot mutant forms of p53. In addition to unfolding several cornerstone findings in the p53 field, these stories also share the fun, the encouragement, the frustration, and the joy of working on p53 with readers, using Arnold J. Levine (whom we fondly call Arnie) as a remarkable example, who is one of the p53 discoverers, including David P. Lane, Lloyd J. Odd, and Pierre May. In part, we would like to take this opportunity to celebrate Arnie’s 80th birthday and his brilliant 40 years’ path on p53 research with a collection of several untold stories. These stories can also enlighten the hearts of youngsters who are interested in science and motivate their enthusiasm about and creativity to biomedical research. As revealed by some of the stories, Arnie often emphasized to his mentees: ‘Just enjoying science’, instead of worrying about its outcomes and being frustrated with bad data or dead-end projects. Under his precious guidance with a great vision on a bigger and long-term picture, many of his mentees have become world-class scientists in different fields as well as in the field of p53. Hence, we hope that while enjoying reading these story-telling essays about p53 and its researchers, our readers, particularly youngsters, could also absorb some scientific nutrients conducive to their future career development in science.

The Review section includes four outstanding, comprehensive, and beautifully written review articles. These essays elegantly describe different aspects of our biochemical, biophysical, molecular, cellular, genetic, physiological, pathological, and translational understandings of p53 and its importance for cancer prevention and cancer therapy. They offer broad views and in-depth analyses on updated p53 research and thus will be breath-taking readings to everyone who is interested in p53 and curious about its research progress in specific areas.

The Perspective section has six prospective essays. These essays articulate how the MDM2–p53 feedback loop is discovered and its application for development of target-based therapy against cancer, and also uncover ‘many faces’ of p53 and scientists’ personal stories on the path of its research. Additionally, these essays offer molecular and biological insights into the anti-cancer activity of wild-type p53 and the oncogenic properties of mutant p53, as well as enticing prospective of conveying the bench knowledge into bed application potentially for anti-cancer therapies. Some key remaining questions on basic and translational p53 research are also raised in these thoughtful writings. Again, some of these essays share personal working and learning experiences with Arnie, and would be jubilant and educational

readings to those who are willing to become scientists or biomedical researchers in the future.

Obviously, this pack of Retrospect, Review, and Perspective is the most recent, though incomplete, collection of untold four-decade stories on p53 as well as its discovery, history, research progress, clinical application, and future. It is not only about the p53 legend itself, but also about the legend scientists who discovered and have devoted their entire life and scientific career to study it. We feel very grateful to all of the writers for their wonderful contributions to this special issue, as without their tremendous effort, it would be impossible to have this great collection of retrospect, review, and prospective essays on p53. We apologize to other outstanding p53 researchers from whom we could not get their own p53 stories partially owing to our inability to get in touch and partially due to space limitation. Although this collection might be imperfect, we are sure that this gap will be eventually made up by other and future p53 researchers at p53’s 50th or 60th anniversary. As for now, let us wish ‘happy 40th birthday, p53, and happy 80th birthday, Arnie!’

